# Matrix Metalloproteinase‐Responsive Hydrogel with On‐Demand Release of Phosphatidylserine Promotes Bone Regeneration Through Immunomodulation

**DOI:** 10.1002/advs.202306924

**Published:** 2024-03-09

**Authors:** Mingjin Zhang, Tingting Yu, Jing Li, Huichun Yan, Liang Lyu, Yi Yu, Gengchen Yang, Ting Zhang, Yanheng Zhou, Xing Wang, Dawei Liu

**Affiliations:** ^1^ Department of Orthodontics Peking University School and Hospital of Stomatology Beijing 100081 China; ^2^ National Center of Stomatology & National Clinical Research Center for Oral Diseases & National Engineering Laboratory for Digital and Material Technology of Stomatology & Beijing Key Laboratory for Digital Stomatology & Research Center of Engineering and Technology for Computerized Dentistry Ministry of Health & NMPA Key Laboratory for Dental Materials Beijing 100081 China; ^3^ Beijing National Laboratory for Molecular Sciences Institute of Chemistry Chinese Academy of Sciences Beijing 100190 China; ^4^ University of Chinese Academy of Sciences Beijing 100049 China

**Keywords:** bone regeneration, hydrogels, macrophage regulation, matrix‐metalloproteinase‐responsive, phosphatidylserine (PS)

## Abstract

Inflammation‐responsive hydrogels loaded with therapeutic factors are effective biomaterials for bone tissue engineering and regenerative medicine. In this study, a matrix metalloproteinase (MMP)‐responsive injectable hydrogel is constructed by integrating an MMP‐cleavable peptide (pp) into a covalent tetra‐armed poly‐(ethylene glycol) (PEG) network for precise drug release upon inflammation stimulation. To establish a pro‐regenerative environment, phosphatidylserine (PS) is encapsulated into a scaffold to form the PEG‐pp‐PS network, which could be triggered by MMP to release a large amount of PS during the early stage of inflammation and retain drug release persistently until the later stage of bone repair. The hydrogel is found to be mechanically and biologically adaptable to the complex bone defect area. In vivo and in vitro studies further demonstrated the ability of PEG‐pp‐PS to transform macrophages into the anti‐inflammatory M2 phenotype and promote osteogenic differentiation, thus, resulting in new bone regeneration. Therefore, this study provides a facile, safe, and promising cell‐free strategy on simultaneous immunoregulation and osteoinduction in bone engineering.

## Graphical abstract

A facile and efficient cell‐free strategy of inflammation‐responsive injectable hydrogel is reported, which for the first time encapsulates, the apoptotic signal molecule, phosphatidylserine into a matrix metalloproteinase‐responsive poly (ethylene glycol) (PEG) network and allows the on‐demand release of therapeutic factor under inflammatory stimulation, aiming to guide long‐term bone regeneration via immune modulation and osteogenic induction.

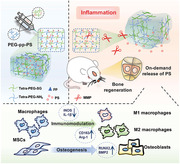

## 1 Introduction

Critical‐sized bone defects caused by trauma, congenital, and oncological diseases affect millions of people physically and psychologically and pose a challenge for effective treatment.^[^ ^1^ ^]^ When the extensive size of osseous defects surpasses their self‐healing capacity, the bone tissue fails to regenerate spontaneously, resulting in bone non‐union and loss of function.^[^ ^2^ ^]^ Various strategies, including autogenous bone grafting, xenogeneic bone grafting, and bone tissue engineering, have been developed to repair and regenerate the bone tissue. Clinical limitations of bone grafting include immune rejection and limited donor tissue availability. Many studies have focused on bone tissue engineering to solve the potential problems associated with the use of scaffolds seeded with cells or cell‐free devices, such as gene therapy and chemical or mechanical signals.^[^ ^3^ ^]^ Cell‐based strategies have been proved effective in bone tissue engineering; however, scaffolds involving seeded cells have some drawbacks, such as restricted cell resources, complicated cell expansion procedures, and relatively low cell survival rates, preventing their clinical translation.^[^ ^4^ ^]^ Compared to the cell‐seeded scaffolds, cell‐free biomaterials exhibit unique advantages, such as low risk of immune rejection and few ethical issues for further clinical translation to bone therapy, by reduplicating and regulating the process of natural self‐healing.^[^ ^5^ ^]^

In the process of bone healing, regulation of inflammation after the pro‐inflammatory stage is an active method to enhance bone regeneration by not only preventing acute inflammation to persist for a long time, but also creating a pro‐regenerative immune microenvironment conductive to osteogenesis.^[^ ^6^ ^]^ Recently, various immunomodulatory biomaterials have been developed for bone‐regeneration medicine.^[^ ^7^ ^]^ For instance, smart hydrogels provide an ideal microenvironment by changing their intrinsic size or structure or controlling the degradation in reaction to the alteration of local inflammation.^[^ ^8^ ^]^ Compared to conventional hydrogels with unstable and arbitrary drug release, inflammation‐responsive hydrogels facilitate the precise delivery of drugs to specific regions to ensure the maximum efficacy of therapeutic factors in regulating the bone defect environment.^[^ ^9^ ^]^ In extensive bone damage, matrix metalloproteinases (MMP) levels are elevated during pathological inflammatory processes and all stages of bone remodeling.^[^ ^10^ ^]^ Accordingly, development of a smart MMP‐responsive hydrogel system may be an effective strategy to release the loaded cargo on demand and treat critical‐sized bone defects.^[^ ^11^ ^]^ Moreover, hydrogels should possess features indispensable for bone tissue engineering, such as adequate mechanical strength, fine cell compatibility, and flexible adaptability to different shapes of defect areas. Our previous work has implied that an injectable tetra‐poly(ethylene glycol) (PEG) hydrogel could fulfill these requirements.^[^ ^12^ ^]^ The network can maintain its inherent properties even in a water environment to adapt to the complicated biological conditions in vivo.^[^ ^13^ ^]^ In view of the highly effective ammonolysis reactions between the active ester groups and amine groups under mild conditions, two components of the injectable tetra‐PEG hydrogel were simultaneously injected into the bone defect area, which rapidly reacted with each other to generate chemical networks within seconds in situ and formed amide bonds with the amine‐terminated protein tissue with high adhesive strength. These features facilitate the simple and rapid synthesis of scaffolds for practical use.^[^ ^14^ ^]^

As ideal drug delivery systems, smart hydrogels can carry the biological products to target areas to perform their immunoregulatory functions. Phosphatidylserine (PS), a small molecule commonly found in the inner membrane of healthy cells, is detected by macrophages after exposure and mediates the M1‐to‐M2 macrophage polarization to reduce early inflammation and enhance tissue repair.^[^ ^15^ ^]^ Reduction in the M1 phenotype alleviates inflammation, and conversion of the M2 phenotype promotes osteogenesis via mesenchymal stem cells (MSCs) and improves bone healing.^[^ ^16^ ^]^ Therefore, PS may be a desirable candidate for encapsulation in scaffolds to promote bone repair. PS‐containing liposomes integrated into traditional scaffolds have shown great potential for bone tissue engineering.^[^ ^17^ ^]^ However, creating a PS‐loaded inflammatory stimulus‐responsive hydrogel system that simultaneously performs multiple functions and exhibits injectability, mechanical properties, and biocompatibility for bone tissue engineering remains an arduous task.

In this study, we constructed an MMP‐responsive injectable hydrogel by connecting an MMP cleavable peptide to a tetra‐PEG network assembled from PS molecules. PS was rapidly delivered to the defect area to regulate macrophage polarization during initial inflammation with high MMP expression, and then sustained at a suitable concentration until the late stage of tissue regeneration. The well‐defined tetra‐PEG network provides sufficient mechanical strength and maintains strong adhesion under complex biological conditions in vivo.^[^ ^13^ ^]^ MMP‐cleavable peptide was integrated to one of the components, PEG‐NH_2_, to form PEG‐pp‐NH_2_ in advance and the two components could rapidly gel after mixture in practical use simplifying the process of production and gelation. Mechanistically, PS not only plays a crucial role in mediating the M1‐to‐M2 polarization in the early stage of inflammation but also enhances osteogenesis to promote bone repair, which exerts significant impact on immune modulation and osteogenic induction for guiding long‐term bone growth. Therefore, our study provides effective injectable and shapeless materials to fill in the gaps of different bone defects and outlines a novel bioactive and easy cell‐free strategy for immune modulation and osteogenic induction to guide long‐term bone growth and healing.

## 2 Results and Discussion

### 2.1 Preparation and Characterization of MMP‐Responsive PEG Hydrogels

**Figure 1:**
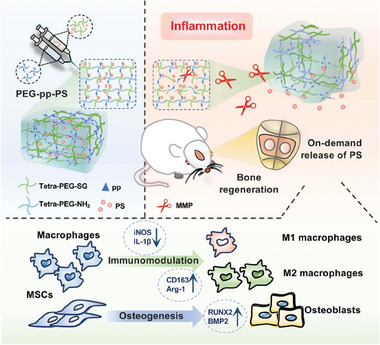
Schematic diagram showing the fabrication procedures of a matrix‐metalloproteinase (MMP)‐responsive phosphatidylserine (PS)‐encapsulated injectable hydrogel (PEG‐pp‐PS) for rat calvaria bone defect regeneration.

**Figure 2:**
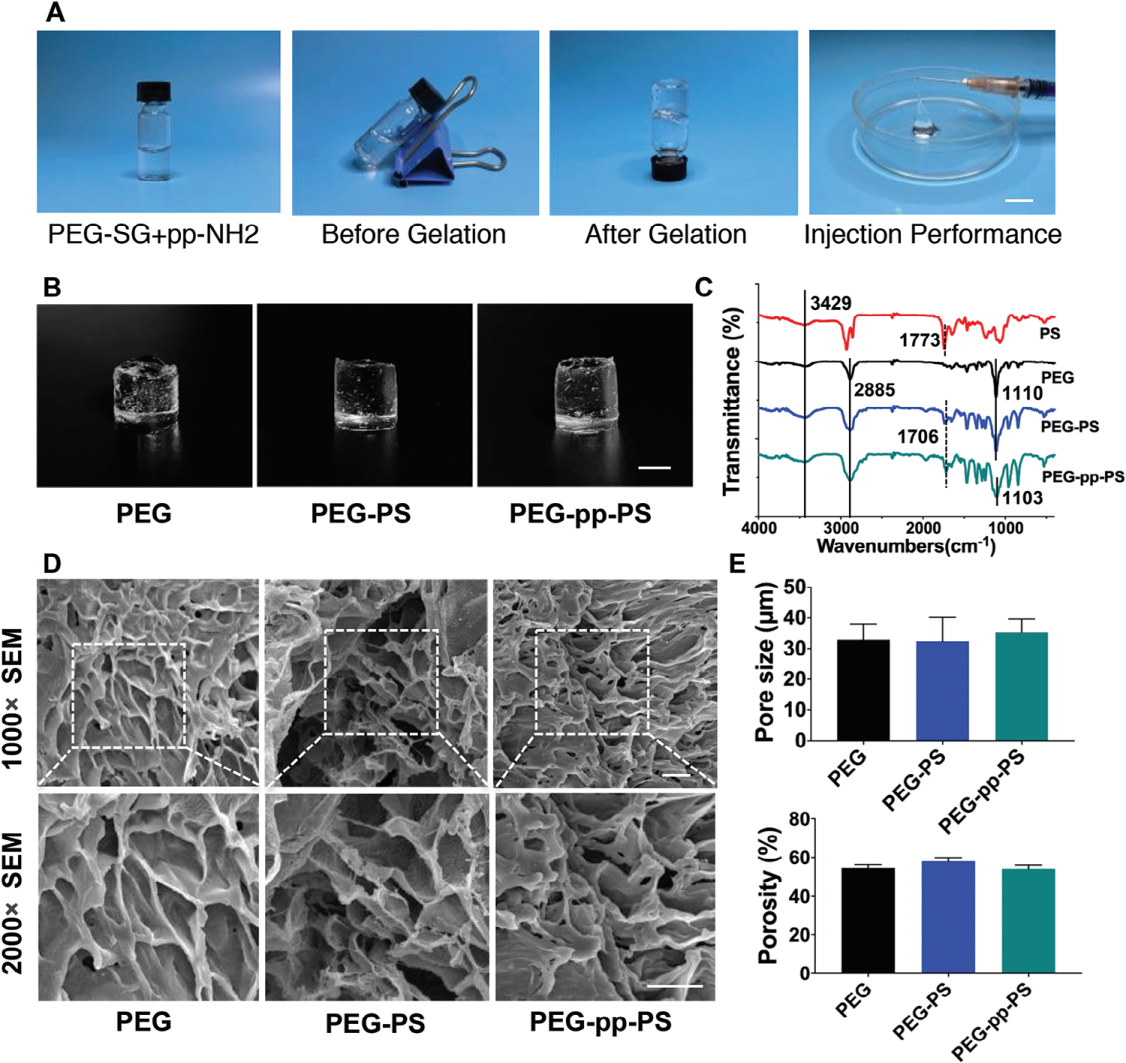
Fabrication and characterization of injectable hydrogel. A) Synthesis procedures for PEG, PEG‐PS, and PEG‐pp‐PS. B) Gross appearance of PEG, PEG‐PS, and PEG‐pp‐PS, scale bar = 0.5 cm. C) FT‐IR spectra of PS, PEG, PEG‐PS, and PEG‐pp‐PS hydrogels. D) SEM images of the inner porous structures of PEG, PEG‐PS, and PEG‐pp‐PS, scale bar = 5 µm. E) Pore size and porosity of PEG, PEG‐PS, and PEG‐pp‐PS hydrogels. Data presented as mean ± standard deviation (SD), n = 3, *p*‐values are calculated using one‐way ANOVA with Tukey's test.

A schematic illustration of the preparation and mechanism of MMP‐responsive PEG‐PS (PEG‐pp‐PS) is shown in **Figure** **1**. Tetra‐PEG‐SG was prepared as previously described.^[^ ^12^ ^]^ MMP2‐cleavable peptides, pp (GPLGIAGQ) were integrated into tetra‐PEG‐NH_2_ to construct tetra‐PEG‐pp‐NH_2_ referring to the previous study.^[^ ^18^ ^]^ Here, tetra‐PEG with a molecular weight of 20 kDa was selected for its mechanical properties and ability to maintain the scaffold structure for a long period. We previously reported that the percentage of drug released from tetra‐PEG is ≈10−15% per day.^[^ ^14^, ^19^ ^]^ Alizarin red S (ARS) staining revealed that PS enhanced osteogenesis in a dose‐dependent manner and facilitated potent osteogenesis of BMMSCs at 50 µg mL^−1^ (Figure S1, Supporting Information). Therefore, PS was encapsulated at a concentration of 500 µg mL^−1^ in the scaffolds to maintain effective drug release. Some PS molecules were distributed in the tetra‐PEG network, while other were connected to the network via chemical linkages due to effective ammonolysis reactions between the active ester groups of the tetra‐PEG‐SG polymer and amine groups of PS under mild conditions. **Figure** **2**A,B showed that the PEG, PEG‐PS, and PEG‐pp‐PS hydrogels exhibited a quick gelation process and easy injectability, with a transparent presentation under gross observation. Fourier‐transform infrared (FT‐IR) spectra was obtained to determine the absorbance peaks of the three hydrogels (Figure 2C). PEG, PEG‐PS, and PEG‐pp‐PS displayed the typical N–H and O–H peaks at 3429 cm^−1^and C–H stretching vibration signals at 2885 cm^−1^. In the spectra of tetra‐PEG and PEG‐PS, the absorbance peak at 1110 cm^−1^ was related to the C–O–C vibration and shifted to 1103 cm^−1^ in the spectra of PEG‐pp‐PS, indicating the formation of chemical bonds between pp and tetra‐PEG. Additionally, the absence of vibration at 1773 cm^−1^ corresponded to O═P–OH in the spectra of PS, and this vibration was located at 1706 cm^−1^ in PEG‐PS and PEG‐pp‐PS, possibly due to the physical chain‐entanglement and hydrogen bonds between PS and tetra‐PEG network. Scanning electron microscopy (SEM) images revealed the highly porous structures of all three hydrogels, which were beneficial for nutrient and metabolic waste exchange and cell growth and proliferation (Figure 2D). We found out that the porous structure of PEG, PEG‐PS, and PEG‐pp‐PS hydrogels did not show significant difference under the same condition of freezing process (Figure 2E). However, the pore size in the SEM images has changed and were not equal to the actual size in vivo due to the freeze‐drying process.

**Figure 3:**
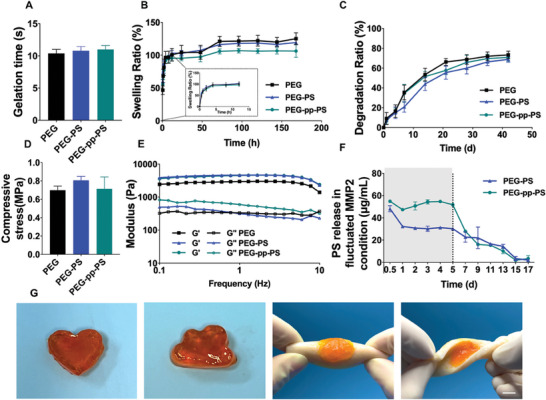
Structural and mechanical properties of injectable hydrogel. A) Gelation time for PEG, PEG‐PS, and PEG‐pp‐PS. B) Swelling, C) degradation profiles, D) and compressive stress of PEG, PEG‐PS, and PEG‐pp‐PS. E) Oscillatory frequency sweep test showing the frequency dependence of storage and loss modulus of PEG, PEG‐PS, PEG‐pp‐PS. F) The dual‐release profile of PS from hydrogels in fluctuated MMP2 condition. G) Photographs showing and that the hydrogel is moldable, extensible, and adhesive; an orange dye was used to stain the hydrogel samples for clear observation, scale bar = 5 mm. Data presented as mean ± SD, *n* = 3, *p*‐values are calculated using one‐way ANOVA with Tukey's test.

To achieve sufficient mechanical strength and an appropriate gelation time for clinical use, 10 wt.% tetra‐PEG‐SG and tetra‐PEG‐NH_2_ were selected for our experiment. The injectable hydrogels gel quickly within 12 s after mixing the components owing to the intrinsic features of the ammonolysis reaction (**Figure** **3**A). No significant differences were observed among the groups, indicating that the PS and the MMP cleavable peptides exerted no influence on the gelation process. Additionally, PEG, PEG‐PS, and PEG‐pp‐PS exhibited similar compressive stresses (Figure 3B). The swelling and degradation behaviors of hydrogels are critical for immediate biological reactions and long‐term drug release of biomaterials in tissue engineering applications.^[^ ^20^ ^]^ Here, PEG, PEG‐PS, and PEG‐pp‐PS reached the swelling equilibrium at 24 h (Figure 3C). The swelling ratio of PEG‐pp‐PS decreased slightly compared to those of the other two scaffolds, probably because of the increasing degree of crosslinking. After 42 days of immersion in PBS, degradation rates of PEG, PEG‐PS, and PEG‐pp‐PS were 73.32 ± 3.07%, 68.85 ± 2.35%, and 70.88 ± 3.26%, respectively (Figure 3D). Rheological behaviors confirmed that the tetra‐PEG hydrogels covered the entire defect area immediately and evenly via simultaneously spraying the two components onto the tissue (Figure 3E). The elastic modulus (*G*’) was larger than the viscous modulus (*G*″), indicating the formation of the hydrogel after the mixing of the two components. All these results imply that PS loading and connection of pp do not significantly change the swelling, degradation characteristics, and mechanical properties of the tetra‐PEG network.

Furthermore, we analyzed the release of PS from PEG‐PS and PEG‐pp‐PS with or without exposure to MMP2 using high‐performance liquid chromatography (HPLC) to reveal the different responsiveness of the hydrogels to inflammation (Figure 3F). The scaffolds were first exposed to 1 µg mL^−1^ of MMP2 in phosphate‐buffered saline (PBS) solution for 5 days and then transferred to immerse into the pure PBS to mimic the fluctuated levels of inflammation in vivo. In the presence of MMP2, PEG‐pp‐PS tended to accelerate the release of PS compared with PEG‐PS, and these two groups followed a similar sustained release pattern after the removal of MMP2 from the solution. The daily release curve demonstrated that the PS release experienced an initial burst on the first day, representing ≈15% in PEG‐PS and 20% in PEG‐pp‐PS, and maintained 5% drug release without MMP2 in both PEG‐PS and PEG‐pp‐PS (Figure S2, Supporting Information). The cumulative release illustrated that 60% of the PS in PEG‐pp‐PS was delivered during the first 5 days in the fluctuating state (Figure S2, Supporting Information). The hydrogels were formed in situ by using a dual syringe to investigate their adaptability and adherence. Figure 3G shows that they could be molded into any shape and remain adhered to the flat porcine skin despite stretching and twisting, indicating that the hydrogels could adapt to the irregular defect area and complex local environment.

### 2.2 PEG‐pp‐PS Enhances Osteogenesis at the Bone Defect Region, Regulating M2 Macrophages Polarization

**Figure 4:**
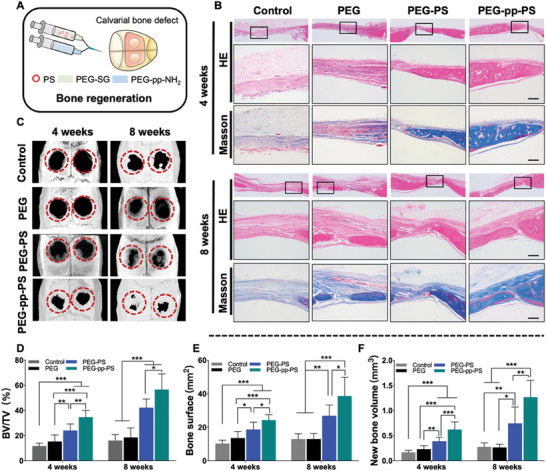
In vivo evaluation of bone regeneration after hydrogel treatment in calvarial bone defect rat model. A) Schematic illustration of calvarial bone defect rat model. B) HE staining and Masson trichrome staining of tissue sections of calvarial defects, scale bar = 200 µm. C) Micro‐CT 3D reconstruction images, and D–F) semiquantitative analysis of BV/TV, bone surface and new bone volume of rat calvarial bone defects implanted with different hydrogels after surgery for 4 and 8 weeks. Data presented as mean ± SD, *n* = 6, *p*‐values are calculated using one‐way ANOVA with Tukey's test, ^*^ *p* < 0.05, ^**^ *p* < 0.01, ^***^ *p* < 0.001.

An SD rat calvarial bone‐defect model was established to investigate the osteogenic ability of PEG‐pp‐PS in vivo (**Figure** **4**A). Full‐thickness calvarial bone defects with a diameter of 5 mm, which are considered as critical‐size defects,^[^ ^21^ ^]^ were prepared, and tetra‐PEG hydrogels were injected in situ simultaneously. The expression of MMP2 in the defect zone was evaluated at 1 week, 4 weeks, and 8 weeks respectively by immunofluorescence staining. As shown in Figure S3 (Supporting Information), MMP2 remained relatively low level in no defect area and excessive amount of MMP2 is released in the early stage of bone defect and increased dramatically at the time point of 1 week. The number of MMP2 positive cells then reduced significantly over time and returned to the normal state at 8 weeks. The fluctuating expression of MMP2 provides a basis for PEG‐pp‐PS to release PS in an on‐demand manner.

Histological staining and micro‐CT were performed to observe the new bone formation in the defect areas of the four groups at 4 weeks and 8 weeks after implantation. H&E staining demonstrated that the PEG‐pp‐PS group showed the greatest bone regeneration compared with the other three groups (Figure 4B). No obvious new bone formation was observed in the control group or PEG groups at 4 weeks. However, the PEG‐PS and PEG‐pp‐PS groups exhibited new bone regeneration around the margins. At 8 weeks after implantation, the defect area was further reduced, and the structure of the de novo bone became more mature compared with at 4 weeks in the PEG‐PS and PEG‐pp‐PS groups. Masson's trichrome staining revealed early bone healing with massive fibrous tissue growth and little bone formation in the control group, whereas a large amount of fibrous bone with blood vessels was observed in the center of the defect area in the PEG‐PS and PEG‐pp‐PS groups (Figure 4B). The micro‐CT results are consistent result with the histological results. Only a limited amount of new bone was formed from the edge of the defect in the control group and PEG group at 4 weeks and 8 weeks, while both the PEG‐PS and PEG‐pp‐PS groups showed stronger osteogenic potential with more new bone regeneration in the margin of the defect at 4 weeks and both in the center and the margin at 8 weeks (Figure 4C). Quantitative data of BV/TV demonstrated that the neo‐bone formation of the PEG‐PS group in 4 and 8 weeks (24.0 ± 4.91% and 42.2 ± 6.40%, respectively) was much greater than the control group (11.7 ± 2.16% and 17.1 ± 5.53%, respectively) and the PEG group (15.2 ± 4.91% and 18.4 ± 6.84%, respectively; Figure 4D). PEG‐pp‐PS group showed the highest osteogenic rate at both time points, with BV/TV of 34.6 ± 4.91% and 56.59 ± 11.36%, respectively, and the differences were statistically significant. Bone surface and volume exhibited similar tendencies among the different groups (Figure 4E,F). The histological and radiographic results showed that PEG‐pp‐PS promoted bone regeneration most prominently among the four groups at different time points.

**Figure 5:**
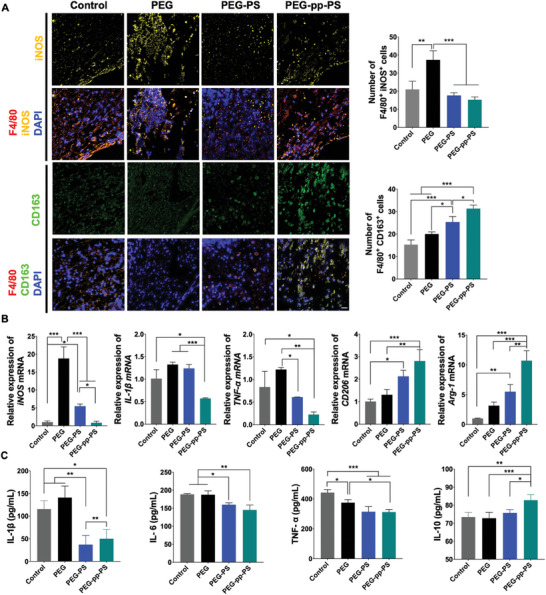
In vivo evaluation of macrophage polarization and inflammation after hydrogel treatment in calvarial bone defect rat model. A) Immunofluorescence shown macrophage polarization of 1 week, scale bar = 20 µm. B) mRNA expression of macrophages shown macrophage polarization. C) ELISA analysis measuring the secretion of inflammatory cytokines (IL‐1β, IL‐6, IL‐10, and TNF‐α). Data presented as mean ± SD, *n* = 3, *p*‐values are calculated using one‐way ANOVA with Tukey's test, ^*^ *p* < 0.05, ^**^ *p* < 0.01, ^***^ *p* < 0.001.

To assess the influence of the hydrogels on the polarization of macrophages in vivo, immunofluorescence staining and quantitative polymerase chain reaction (qPCR) were performed to examine the expression of relevant genes in the calvarial bone site at the time point of 1 week. Compared with the control group, the number of F4/80‐positive inducible nitric oxide synthase (iNOS)‐positive M1 macrophages increased prominently in the PEG group, and the numbers in the PEG‐PS and PEG‐pp‐PS groups remained low. The number of F4/80‐positive CD163‐positive M2 macrophages increased significantly in the PS‐encapsulated scaffolds. Moreover, the increase was the most significant in the PEG‐pp‐PS group, which indicated that more PS was released from PEG‐pp‐PS in response to inflammation and was taken up by M1 macrophages. M1 polarization was inhibited at the acute inflammatory stage, and more macrophages transformed into M2 macrophages to exert anti‐inflammatory effects (**Figure** **5**A). Figure 5B shows the relative expression levels of macrophage polarization genes. Compared with the control group without any implants, the M1‐macrophage marker iNOS was highly expressed in both the PEG and PEG‐PS groups, which might be due to the material‐induced immune response. However, interestingly, the levels of iNOS were not significantly different between the PEG‐pp‐PS and the control groups, indicating that the MMP‐responsive hydrogel could regulate the immune environment more efficiently. The similar tendency was detected in M1‐macrophage related gene interleukin (IL)−1β and tumor necrosis factor (TNF)‐α. The qPCR results also revealed that the M2‐macrophage related gene, CD206, and arginine (Arg)−1 levels were significantly upregulated in the PEG‐pp‐PS group than in the other three groups.

Macrophage polarization can affect the production of cytokines and consequently regulate the microenvironment in situ. Therefore, to confirm how the hydrogels might influence the secretion of cytokines, enzyme‐linked immunosorbent assay (ELISA)_of inflammation‐related cytokines was performed at the protein expression level at 1 week (Figure 5C). The secretion of pro‐inflammatory cytokines IL‐1β, TNF‐α, and IL‐6 showed the similar tendency as the mRNA expressions. The anti‐inflammatory cytokine, IL‐10, was significantly upregulated only in the PEG‐pp‐PS group. These results suggest the downregulation of M1 macrophage expression 1 week after PEG‐pp‐PS implantation and the induction of macrophage transformation to the M2 phenotype at the early stage of the bone repair process.

### 2.3 PEG‐pp‐PS Regulates Macrophages Polarization and Osteogenesis In Vitro

**Figure 6:**
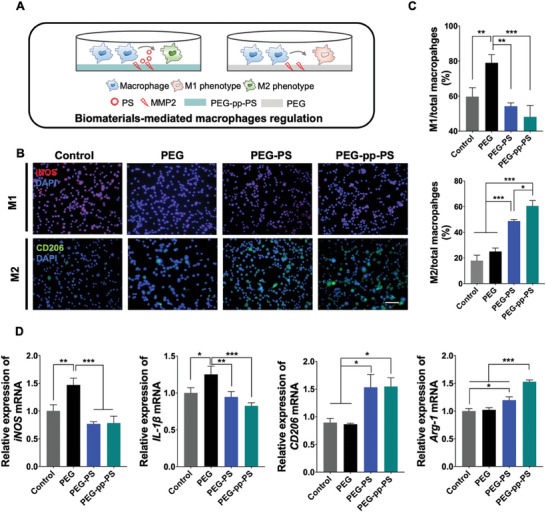
The effect of scaffold treatment on the polarization and inflammation‐related gene expression of macrophage in vitro. A) Schematic illustration. B) Fluorescence microscopy images of immunofluorescent staining of iNOS and CD206, scale bar = 200 µm. C) The ratio of M1 and M2 macrophages to total macrophages in fluorescence microscopy images. D) mRNA expression of inflammation and polarization genes of macrophages in the MMP2 condition treated with hydrogel loaded with PS. Data presented as mean ± SD, *n* = 3, *p*‐values are calculated using one‐way ANOVA with Tukey's test, ^*^ *p* < 0.05, ^**^ *p* < 0.01, ^***^ *p* < 0.001.

Next, we confirmed whether the scaffolds regulated macrophages and promoted bone regeneration in vivo and determined the underlying mechanism. First, we performed an in vitro study via the co‐culture of mouse macrophages (RAW264.7) with different tetra‐PEG hydrogels in a cell culture medium containing 1 µg mL^−1^ of MMP2 to mimic the inflammatory conditions (**Figure** **6**A). Immunofluorescence staining, quantitative reverse transcription (qRT)‐PCR, and flow cytometry were also performed to assess macrophage polarization. Figure 6B,C demonstrate a similar trend in macrophages polarization as that observed in vivo via immunofluorescence staining. PEG‐pp‐PS and PEG‐PS treatment decreased the ratio of the iNOS‐positive M1 macrophages to total macrophages compared to that in the PEG group, whereas PEG‐pp‐PS increased the ratio of the CD206‐positive M2 macrophages to total macrophages remarkably. qRT‐PCR (Figure 6C) revealed that, compared with the control group, expression levels of the M1 markers, iNOS and IL‐1β, were increased in the PEG group and slightly decreased in the PEG‐pp‐PS and PEG‐PS groups. In contrast, expression levels of M2 markers CD206 and Arg‐1, were increased in the PEG‐pp‐PS and PEG‐PS groups. However, no significant difference in the M1 macrophage ratio was observed between the PEG‐pp‐PS and control groups. This may be explained by the fact that the anti‐inflammatory effect of PS is cancelled out by the biological response of the cells to the hydrogels. The same trend was observed in flow cytometry (Figure S4, Supporting Information), which revealed higher expression levels of CD206 (19.3%) in the PEG‐pp‐PS group than in the control (11.7%), PEG (9.4%), and PEG‐pp‐PS (13.2%) groups. Expression level of CD86, the M1 macrophage representative marker, showed a similar trend as the results of qRT‐PCR. These results indicated that PEG‐pp‐PS transforms macrophages into the M2 phenotype more effectively than the hydrogels loaded with PS under inflammatory conditions in vitro. In recent times it is discovered that macrophages exist more complicated phenotypes between stereotypical M1 and M2 populations; specifically, M2a and M2c can be observed locally at the injury site during bone regeneration.^[^ ^22^ ^]^ M2a macrophages (prohealing) could upregulate the surface marker CD206 and M2c macrophages (promodeling) could be distinguished by expression of CD163.^[^ ^23^ ^]^ In our study, images of immunofluorescent staining have displayed that both CD206 positive and CD163 positive M2 macrophages increased significantly in the PS‐encapsulated scaffolds which implied that PS provided more possibilities of M2 macrophages polarization to promote bone regeneration in different ways unlike the utilization of single type of cytokine.

**Figure 7:**
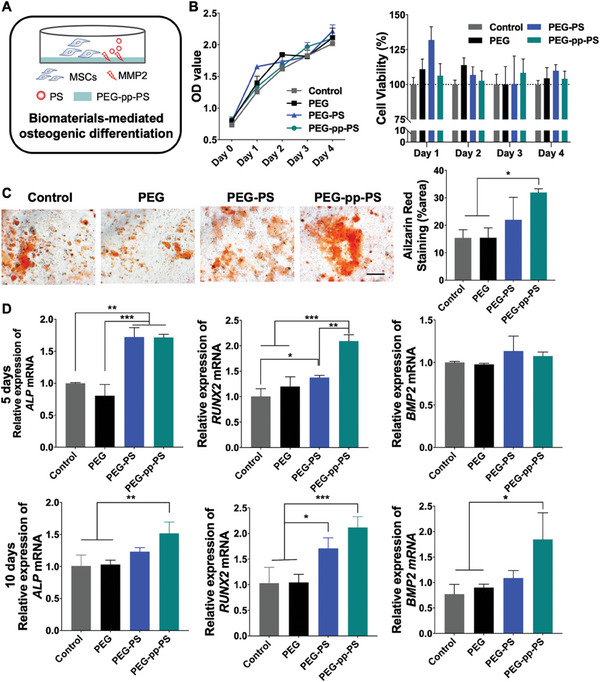
Cytotoxicity and osteogenic potential of MSCs after hydrogel treatment in vitro. A) Schematic illustration. B) CCK‐8 assay of MSCs proliferation and viability after co‐culture for 24, 48, 72, and 96 h. C) ARS staining of MSCs co‐cultured with different scaffolds, scale bar = 500 µm. D) mRNA expression of osteogenesis genes of MSCs in the MMP2 condition treated with hydrogel loaded with PS. Data presented as mean ± SD, *n* = 3, *p*‐values are calculated using one‐way ANOVA with Tukey's test, ^*^ *p* < 0.05, ^**^ *p* < 0.01, ^***^ *p* < 0.001.

During pathological bone injury, macrophages not only contribute to the inflammatory reaction but also initiate the recruitment of MSCs, which are mobilized from the bone marrow to the lesion site and subsequently differentiate into osteoblasts for bone repair.^[^ ^24^ ^]^ Apoptotic bodies derived from MSCs could maintain MSCs homeostasis to enhance bone repair.^[^ ^25^ ^]^ We inferred that PS may mimic apoptotic signals and played a role similar as apoptotic bodies in tissue repair and regeneration. In our pilot experiment, PS promoted osteogenesis in an appropriate concentration (Figure S1, Supporting Information). Immunofluorescence staining of rat calvarial bone defects revealed more cells positive for the osteogenic markers, RUNX family transcription factor 2 (RUNX2), and alkaline phosphatase (ALP), in the PEG‐pp‐PS group at 4 and 8 weeks (Figure S5, Supporting Information). To determine whether the PS‐encapsulated hydrogels activated cells with osteogenic potential in situ and promoted endogenous bone formation in vitro, we directly co‐cultured the mouse bone marrow mesenchymal stem cells (BMMSCs) with different groups of hydrogels in a cell culture medium containing 1 µg mL^−1^ of MMP2 (**Figure** **7**A). Cell proliferation assays were performed at 24, 48, 72 and 96 h using the cell counting kit‐8 (CCK‐8). Slight disparities in BMMSC viability were observed among the four groups, but they were not significant, indicating that neither PEG nor PS‐encapsulated PEG exhibited cytotoxicity (Figure 7B). Then, ARS staining was performed to evaluate the osteogenic differentiation of MSCs after the implantation of BMMSCs into the biomaterials (Figure 7C). PEG‐pp‐PS group exhibited the most mineralized nodules and a significantly higher ratio of positively stained areas than the control and PEG groups. Additionally, qRT‐PCR was used to evaluate the relative mRNA expression levels of the osteogenic marker genes, including RUNX2, ALP, and bone morphogenetic protein 2 (BMP2), at days 5 and 10, respectively (Figure 7D). Expression levels of RUNX2 and BMP2 were higher in the PEG‐PS and PEG‐pp‐PS groups than in the control and PEG groups on day 5. However, no significant differences of BMP2 expression levels were found among the four groups during the early stage of osteogenesis. Interestingly, after 10 days of osteogenesis, the expression levels of all three osteogenic markers (ALP, RUNX2, and BMP2) increased significantly in the PEG‐pp‐PS group, implying that the release of a large amount of PS from the PEG‐pp‐PS network effectively promotes osteogenic differentiation more effectively under inflammatory conditions. Therefore, PS, a kind of small molecule abundant in the body, may be a promising and safe candidate to regulate the immune microenvironment and promote bone repair without any complex biological side effects. In summary, PEG‐pp‐PS is a promising biomaterial that not only regulates macrophage polarization to protect the tissues from persistent inflammation but also enhances osteogenic differentiation at all stages of bone repair.

## 3 Conclusion

In summary, we developed an MMP‐responsive tetra‐PEG gel loaded with PS to regulate drug release according to the inflammatory microenvironment. The developed PEG‐pp‐PS hydrogel scaffold not only exhibited desirable mechanical properties and cell compatibility but also delivered PS precisely, conforming to the different stages of tissue repair. The key therapeutic factor, PS, has great osteogenic potential owing to its synergistic action in regulating M2 macrophage polarization and promoting osteogenic differentiation, thereby, facilitating bone regeneration. This study provides a new and promising cell‐free therapeutic strategy for bone tissue engineering.

## 4 Experimental Section

Detailed experimental section can be found in the Supporting Information.

## Conflict of Interest

The authors declare no conflict of interest.

## Supporting information

Supporting Information

## Data Availability

The data that support the findings of this study are available from the corresponding author upon reasonable request.
