# Supplementary material for: Matrix Metalloproteinase‐Responsive Hydrogel with On‐Demand Release of Phosphatidylserine Promotes Bone Regeneration Through Immunomodulation
Source: Adv Sci (Weinh). 2024 Mar 9;11(20):2306924. doi: 10.1002/advs.202306924 (PMC11132073; doi:10.1002/advs.202306924)
Supplement: Supplementary file 1 — Supporting Information [file ADVS-11-2306924-s001.pdf]

ADVANCED SCIENCE
Open Access

Supporting Information

for *Adv. Sci.*, DOI 10.1002/advs.202306924

Matrix Metalloproteinase-Responsive Hydrogel with On-Demand Release of Phosphatidylserine Promotes Bone Regeneration Through Immunomodulation

Mingjin Zhang, Tingting Yu, Jing Li, Huichun Yan, Liang Lyu, Yi Yu, Gengchen Yang, Ting Zhang, Yanheng Zhou, Xing Wang* and Dawei Liu**

Supporting Information

Matrix Metalloproteinase-Responsive Hydrogel with On-Demand Release of Phosphatidylserine Promotes Bone Regeneration through Immunomodulation

Mingjin Zhang†, Tingting Yu†, Jing Li, Huichun Yan, Liang Lyu, Yi Yu, Gengchen Yang, Ting Zhang, Yanheng Zhou, Xing Wang*, Dawei Liu**

† These authors contributed equally to this work.

Mrs. M. Zhang, Dr. T. Yu, Dr. J. Li, Mrs. H. Yan, Mr. L. Lyu, Mr. Y. Yu, Mr. G. Yang, Mrs. T. Zhang, Prof. Y. Zhou, Prof. D. Liu
Department of Orthodontics, Peking University School and Hospital of Stomatology, Beijing, 100081, China.
E-mail: yanhengzhou@vip.sina.com, liudawei@bjmu.edu.cn

Mrs. M. Zhang, Dr. T. Yu, Dr. J. Li, Mrs. H. Yan, Mr. L. Lyu, Mr. Y. Yu, Mr. G. Yang, Mrs. T. Zhang, Prof. Y. Zhou, Prof. D. Liu
National Center of Stomatology & National Clinical Research Center for Oral Diseases & National Engineering Laboratory for Digital and Material Technology of Stomatology & Beijing Key Laboratory for Digital Stomatology & Research Center of Engineering and Technology for Computerized Dentistry Ministry of Health & NMPA Key Laboratory for Dental Materials, Beijing, 100081, China.

Dr. X. Wang
Beijing National Laboratory for Molecular Sciences, Institute of Chemistry, Chinese Academy of Sciences, Beijing 100190, China.
E-mail: wangxing@iccas.ac.cn

Dr. X. Wang
University of Chinese Academy of Sciences, Beijing 100049, China.

Experimental Section

Materials

Tetra-PEG-amine (Tetra-PEG-NH_2, M_w = 20 kDa) and Tetra-PEG-SG (M_w = 20 kDa) were purchased from SINOPEG (Xiamen, China). PS (P0474) was purchased from Sigma-Aldrich (St. Louis, MO, USA). 1-(3-(dimethylamino) propyl)-3-ethylcarbodiimide hydrochloride (EDCI) and N-hydroxyl succinimide (NHS) were

purchased from Energy Chemical. MMP2 was obtained from Proteintech. The MMP2-cleavable peptides, pp (GPLGIAGQ), were synthesized by China Peptides (Shanghai, China). Infrared (IR) grade potassium bromide was purchased from Aladdin (Shanghai, China). Ethanol, isopropanol, chloroform, and glutaraldehyde were purchased from Beijing Chemworks (Beijing, China). Six-week-old female C57BL6 mice and six-week-old female SD rats were purchased from SiPeiFu Biotechnology Co., Ltd. (SPF; Beijing, China).

Synthesis, preparation of PS-Encapsulated tetra-PEG-SG hydrogel (PEG, PEG-PS, PEG-pp-PS)

All solutions were prepared in ultra-pure water at room temperature. Tetra-PEG-NH_2 (10 wt%) was dissolved in a 1 mL of bottle to form a precursor solution, and tetra-PEG-SG (10 wt%) was dissolved in in another bottle. Same volumes of these two precursor solutions were simultaneously mixed or injected together via vortexing and then stood at room temperature to allow gelation.

When preparing PEG-PS hydrogels, PS (500 μg mL^{-1}) was dissolved in the sample bottle, precursor molecules of tetra-PEG-SG (10 wt%) were then added into the PS solution. An equivalent volume of tetra-PEG-NH_2 solution (10 wt%) was added to the PS@tetra-PEG-SG solution. The solution was thoroughly mixed via vortexing and then stood at room temperature to allow gelation. PS was not required for the pure PEG hydrogel.

When preparing PEG-pp-PS hydrogels, the MMP2-cleavable peptide, pp (5 mg) was firstly reacted with the tetra-PEG-NH_2 solution (10 wt%) in the NHS in the presence of a trace amount of EDCI at room temperature overnight. The crude was purified by the dialysis (MWCO 2000 Da) against water for 48 h, followed by the freeze-dry, affording the PEG-pp-NH_2 as white powder. An equivalent volume of tetra-PEG-NH_2 solution (10 wt%) was added to the PS-tetra-PEG-SG solution.

Flourier transform infrared spectroscopy (FT-IR)

A small amount of PS and freeze-dried gels were mixed with IR-grade potassium bromide, ground to a fine powder, dried, and compacted into disks. In the wavenumber

range of 4000-400 cm^{-1}, 32 scans were carried out with a resolution of 2 cm^{-1} using FT-IR (Bruker, Karlsruhe, Germany).

Scanning electrical microscopy (SEM)

Briefly, the hydrogel samples were fixed using 2.5% glutaraldehyde, gradient ethanol dehydrated, and lyophilized for SEM (Hitachi, Tokyo. Japan) observation. SEM images of PEG (n = 3), PEG-PS and PEG-pp-PS (n = 3) were acquired.

Measurement of the gelation time

The gelation time was measured by the vial tilting method at the room temperature. The different components of PEG, PEG-PS and PEG-pp-PS dissolved in PBS (pH=7.4) were mixed respectively in the sample vials. The time at which there was no flow upon inverting the vial was regarded as the gelation time.

Swelling and degradation ratios

The initial wet weight, W_0, of freeze-dried PEG samples was recorded. Then, samples were immersed in PBS at 37 °C, and the PBS was replaced every 2 days. In the swelling study, samples were weighed after 0.5, 1, 2, 4, 8, 12, 24, 48, 72, 96, 120, 144 and 168 h, and their weights were recorded as W_t. The swelling ratio (%) of the samples was calculated using following formula:

$$\text{Swelling Ratio (\%)} = (W_t - W_0)/ W_0 \times 100\%$$

When measuring degradation, the samples were rinsed, lyophilized, and weighed after 0 (W_0), 1, 4, 7, 14, 21, 28, 35, and 42 days, and the weights were recorded as W_t. The degradation ratio (%) of the samples was calculated using the following formula:

$$\text{Degradation Ratio (\%)} = (W_0 - W_t)/ W_0 \times 100\%$$

Compression stress

PEG, PEG-PS and PEG-pp-PS were prepared in a container with a diameter of 10 mm and a height of 7.5 mm. All the samples were tested by Universal Testing Systems (Instron, MA, USA) at a compression rate of 2 mm min^{-1}.

Rheological tests

Rheological behaviors were conducted on a Thermo Haake Rheometer (Newington, NH, United States). During the experiments, the hydrogels were set on a

cone-parallel plate geometry (35 mm of diameter) at a gap of 2.5 mm. All the samples were measured at 25 °C in a frequency range of 100–0.1 rad s^{-1}.

In vitro drug release from the hydrogels

The two kinds of hydrogel samples (PEG-PS and PEG-pp-PS) were prepared in a container with the diameter of 10 mm and height of 2mm, and all the hydrogel samples were immersed into the PBS with MMP2 (1 μg mL^{-1}) at the first 5 days and the pure PBS at the following time points. The solutions were collected at the time points of 12 h, 1, 2, 3, 4, 5, 7, 9, 11, 13, 15 and 17 days. The PS concentration was measured by high performance liquid chromatography (HPLC) on a Thermo Ultimate 3000 system with UV detection at 203 nm. A mixture of acetonitrile and isopropanol (v/v=7:3) was used as mobile phase at a flow rate of 1.0 mL min^{-1}.

Surgical procedure of rat calvarial bone defect model

The animal experiments were approved by Peking University Biomedical Ethics Committee (approval number LA2021002). Critical-sized bone defects with 5 mm diameter were prepared in female 6-8 weeks Sprague−Dawley rat calvarial bone. The PEG, PEG-PS and PEG-pp-PS scaffolds were injected and coagulated in the defects. The control group was without any implants. To assess the bone regeneration potential of the hydrogels, the rats were sacrificed after implantation for 4 or 8 weeks, and the calvarial bones were removed and fixed in 10% formalin. To assess the macrophage reaction to the hydrogels, the rats were sacrificed after implantation for 1 week, and the calvarial bones were removed.

Micro-CT scanning and analysis

To analyze neo-bone formation, the calvarial bone specimens were scanned using a Skyscan 1174 micro-CT system (Bruker, Belgium) at a resolution of 20 μm. The acquired axial images were exported into a NRecon and CTvox software for 3-dimensional reconstruction. Bone volume/total volume (BV/TV) ratio, bone volume and bone surface were calculated using a CTAn (Burker) software.

Histomorphometry analysis

After micro-CT scanning, the samples were decalcified in 10%

ethylenediaminetetraacetic acid for 4 weeks, dehydrated in ethanol, and embedded in paraffin. Consecutive 4 μm-thick horizontal sections were obtained from the defect area and then stained with H&E and Masson Staining Kits (Solarbia) for new bone and remnant scaffold assessment. Each slide was observed using a Zeiss light microscopy at the defect area.

Immunofluorescence staining

Briefly, specimens were immersed in antigen retrieval solution for 30 min, blocked for 60 min with 5% bovine serum albumin (BSA), and subsequently incubated with primary antibodies against F4/80 (Cat. sc-52664, Santa Cruz Biotechnology) for a macrophage marker, CD163 (Cat. 16646-1-AP, Proteintech Group, Rosemont, IL, USA) and CD206 (Cat. 60143-1-Ig, Proteintech Group, Rosemont, IL, USA) for M2 markers, iNOS (Cat. 18985-1-AP, Proteintech Group, Rosemont, IL, USA) for a M1 marker, and RUNX2 (Cat. sc-101145, Santa Cruz Biotechnology) and ALP (Cat. sc-365765, Santa Cruz Biotechnology) for osteogenic markers at 1:100 dilution overnight at 4 °C. For *in vivo* studies, macrophages on the different-coated coverslip were fixed in 4% paraformaldehyde, permeabilized with 0.25% Triton-X, and blocked by 5% BSA. Subsequently, the primary antibodies against CD206, and iNOS with 1:100 dilution were dropped onto coverslips and incubated overnight at 4 °C.

After rinsing thoroughly in PBS, the horseradish peroxidase-conjugated secondary antibodies (Zhongshan Golden Bridge Biotechnology) were dropped onto slides for 1 h. The mounting medium with 4, 6-diamidino-2-phebylindole (DAPI; Zhongshan Golden Bridge Biotechnology, Beijing, China) was used to seal the sections. Each group is composed of more than three slides, and each slide was observed by laser scanning confocal microscopy with 20× and 40× objective lenses (LSM 510; Zeiss, Germany), and images were captured by software (LSM 5 Release 4.2 Software). Quantitative analysis was performed using Image J (NIH, Bethesda, MD, USA).

Cytokine measurements by enzyme-linked immunosorbent assay

The supernatants of rat calvarial bone defect areas were collected at one week and stored at −80 °C before use. The secretion of major M1- and M2-associated cytokines,

IL-1β, TNF-α, IL-6, and IL-10, was examined with ELISA kits (R&D systems) following the manufacturer's guidance.

In vitro cell culture and induction

RAW264.7 were cultured RMPI 1640 containing 10% fetal bovine serum (20%; Gibco) and penicillin/streptomycin (1%; Gibco). The isolation of mBMMSCs were performed as described in our previous studies[1]. After 2 days, nonattached cells were discarded, and adherent cells were cultured for 14 days in mBMMSC growth medium. The mBMMSC growth medium comprised complete alpha minimum essential medium (Biological Industries, Beit Haemek, Israel) supplemented with 20% fetal bovine serum (20%; Gibco), L-glutamine (2 mM; Gibco), 2-mercaptoethanol (55 μM; Gibco), and penicillin/streptomycin (1%; Gibco).

BMMSCs at passage 2 were prepared for osteogenic induction, and the osteogenic medium was used for BMMSC culture which contained complete culture medium plus ascorbic acid (50 mg L^{-1}; Sigma-Aldrich, MO, USA), β-glycerophosphate (10 mM; Sigma-Aldrich), and dexamethasone (10 nM; Sigma-Aldrich).

All the cells were cultured at 37°C and 5% CO_2.The medium was refreshed per three days, and then colony-forming attached cells were passaged once for further experimental use.

Quantitative real-time reverse transcription PCR (qRT-PCR)

Total RNA extraction was conducted following the manufacturer's protocol using the TRIzol Reagent (Invitrogen, Waltham, MA, USA). The RNA concentration was determined using a NanoDrop 8000 spectrophotometer (Nanodrop Technologies, Wilmington, DE, USA). Total RNA (1 μg per sample) was reverse-transcribed to cDNA using ReverTra ACE qPCR RT Master Mix (TOYOBO, Osaka, Japan) following manufacturer's protocol. The cDNA was used as a template in a qPCR reaction, performed using FastStart Universal SYBR Green Master (Roche, Basel, Switzerland) on an ABI Prism 7500 Real-Time PCR System (Applied Biosystem, Foster City, CA, USA). Target gene expression was normalized to that of GAPDH (encoding glyceraldehyde-3-phosphate dehydrogenase). The result was analyzed using the $2^{(-\Delta\Delta CT)}$

method[2]. The primer sequences are listed in **Table S1**.

Table S1. Primer sequences of target genes for qPCR.

Genes	Forward (5'-3')	Reverse (3'-5')
Rat-GAPDH	TGTTCCAGTATGACTCTACCCAC	CATTTGATGTTAGCGGGATCTCG
Rat-iNOS	TCTTGGAGCGAGTTGTGGATTGTTC	AGTGATGTCCAGGAAGTAGGTGAGG
Rat-IL-1β	AATCTCACAGCAGCATCTCGACAAG	TCCACGGGCAAGACATAGGTAGC
Rat-TNF-α	CCACGCTCTTCTGTCTACTGAACTTC	AGATGATCTGAGTGTGAGGGTCTGG
Rat-CD206	GACAGACGGACGAGGAGTTCATTATAC	CCACCAATCACAACAACACAGTCAAC
Rat-Arg-1	AGAGGAGGTGACTCGTACTGTGAAC	TCTGGCTTATGATTACCTTCCCGTTTC
Mouse-GAPDH	AGGTCGGTGTGAACGGATTTG	GGGGTCGTTGATGGCAACA
Mouse-iNOS	GTTCTCAGCCCAACAATACAAGA	GTGGACGGGTCGATGTCAC
Mouse-IL-1β	GAAATGCCACCTTTTGACAGTG	TGGATGCTCTCATCAGGACAG
Mouse-CD206	CCGACATGCCAGGACGAAAG	AGGAGTTGTTGTGGGCTCTG
Mouse-Arg-1	CTCCAAGCCAAAGTCCTTAGAG	AGGAGCTGTCATTAGGGACATC
Mouse-ALP	ATGGTAACGGGCCTGGCTACA	AGTTCTGCTCATGGACGCCGT
Mouse-	CCTGAACTCTGCACCAAGTCCT	TCATCTGGCTCAGATAGGAGGG

RUNX2		
Mouse-BMP2	TCTTCCGGGAACAGATACAGG	TGGTGTCCAATAGTCTGGTCA

Cytotoxicity assays

Cell Counting Kit-8 (CCK-8) (Dojindo, Kumamoto, Japan) assays (n = 6) were applied following manufacturer's protocol. BMMSCs were seeded in a 96-well plate at 5×10^3 cells per well. After incubation for 24 h, PEG, PEG-PS, PEG-pp-PS was added in the related corresponding groups respectively for further incubation. OD values (450 nm) of the experimental group, negative control group, and background were recorded as OD_E, OD_{NC} and OD_B, respectively. Cell viability (%) was defined using the following formula:

$$\text{Cell Viability (\%)} = (OD_E - OD_B)/\ (OD_{NC} - OD_B) \times 100\%$$

Alizarin Red S (ARS) staining

After 21-days of osteogenic induction, mBMMSCs were fixed by 4% of paraformaldehyde (PFA) at ambient temperature for 15 min and then rinsed with phosphate-buffered saline (PBS) three times. BMMSCs were stained with 2% of ARS (Solarbio, Beijing, China) dye for 20 min at room temperature and then rinsed with ddH_2O three times. Stained BMMSCs were air-dried and photographed. Image J was used to measure the positively staining areas semi-quantitatively (n = 3).

Analysis of macrophage phenotype markers by flow cytometry

The macrophages samples using different treatments were firstly collected and washed with PBS. To detect the expression of M1 macrophages, the macrophages samples were incubated with anti-mouse CD86-APC (BD Bioscience, USA) for 15 min incubating in dark at room temperature. In addition, the anti-mouse CD206-APC (BD Bioscience, USA) were used to detect the expression of M2 macrophages. After incubating, flow cytometry was used to analysis the expression of macrophage phenotype with a BD FACS Calibur flow cytometer (BD Biosciences, USA).

Statistics analysis

Data processing and analysis were conducted with SPSS software (ver. 13.0; SPSS Inc., USA) and GraphPad Prism (8.1 version; GraphPad Software, CA, USA). All values calculated are expressed as the mean and standard deviation (mean ± SD), representing 3-6 independent experiments. Comparison of two groups was executed by independent unpaired two-tailed Student's t-tests. For difference analysis among more than two groups, one-way ANOVA with Tukey's test was conducted. $p < 0.05$ was considered to be significant (*$p < 0.05$, **$p < 0.01$, ***$p < 0.001$).

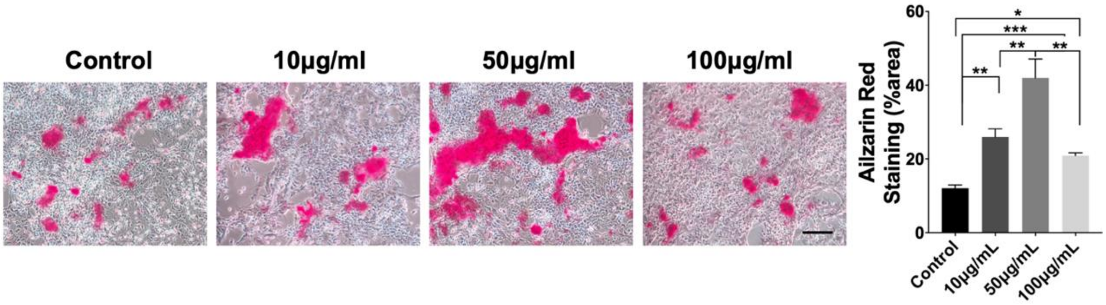

Figure S1. ARS staining shown PS enhanced MSCs osteogenic differentiation in vitro in a dose-dependent manner. Data presented as mean ± SD, n = 3, p-values are calculated using one-way ANOVA with Tukey's test, $^{*}p < 0.05$, $^{**}p < 0.01$, $^{***}p < 0.001$.

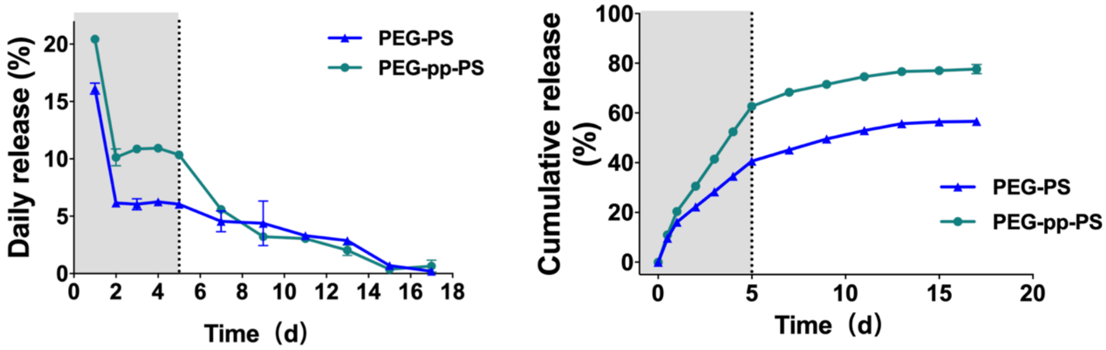

Figure S2. Daily release and cumulative release curve of PS from hydrogels in the inflammation mimicking environment. Data presented as mean ± SD, n = 3.

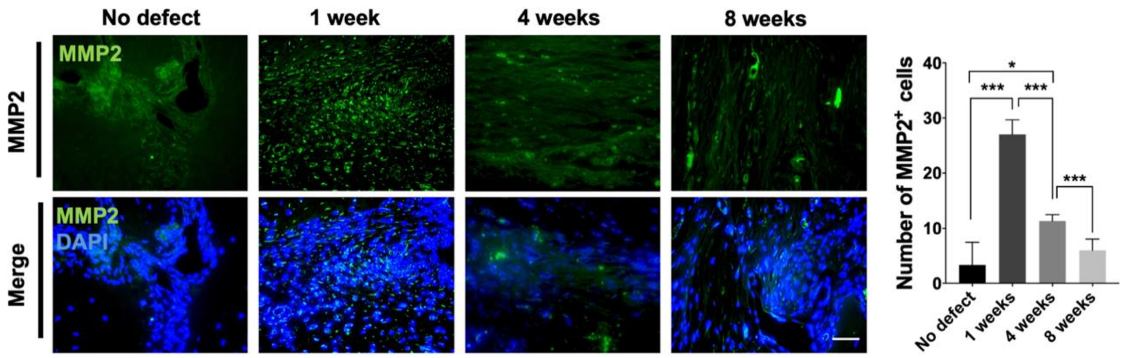

Figure S3. Immunofluorescence shown fluctuated concentrations of MMP2 in animal model. Data presented as mean ± SD, n = 3, p-values are calculated using one-way ANOVA with Tukey's test, $^{*}p < 0.05$, $^{**}p < 0.01$, $^{***}p < 0.001$.

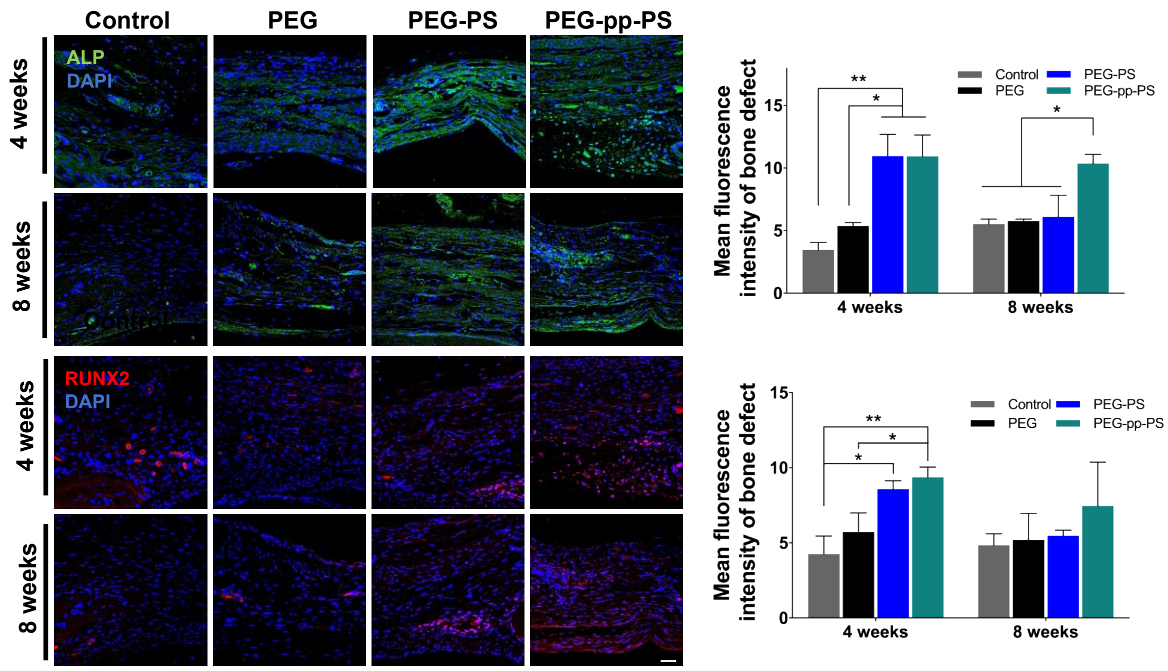

Figure S4. Immunofluorescent staining images of RUNX2 and ALP in calvarial bone defect areas in 4 and 8 weeks, scale bar = 50 μm. Data presented as mean ± SD, n = 3, p-values are calculated using one-way ANOVA with Tukey's test, $^{*}p < 0.05$, $^{**}p < 0.01$.

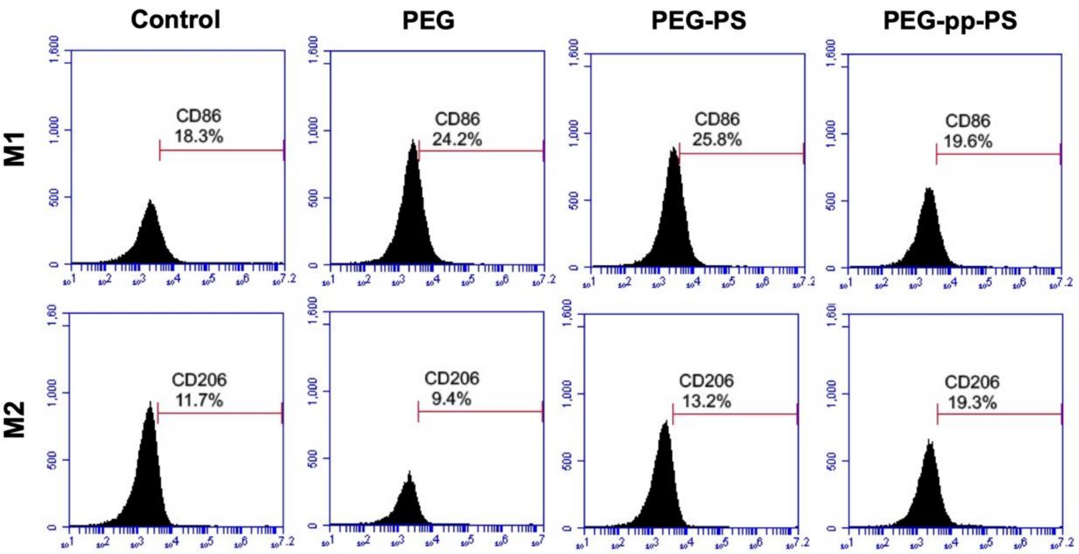

Figure S5. M1 and M2 macrophage identification from LPS-treated RAW264.7 by FC.

References

[1] Liu D, Kou X, Chen C, Liu S, Liu Y, Yu W, Yu T, Yang R, Wang R, Zhou Y, Shi S, *Cell research* **2018,** *28*, 918.

[2] Livak KJ, Schmittgen TD, *Methods* **2001,** *25*, 402.
